# Acute efficacy of a focal dual-energy contact force-sensing catheter vs. standard radiofrequency for anatomical ablation of persistent atrial fibrillation: a pilot study

**DOI:** 10.1093/europace/euag196

**Published:** 2026-08-19

**Authors:** Francesco Notaristefano, Nicolas Johner, Geoffroy Ditac, Allan Plant, Laurens Verhaeghe, John L Fitzgerald, Kinan Kneizeh, Karim Benali, Konstantinos Vlachos, Cinzia Monaco, Marine Arnaud, Benjamin Sacristan, Jan Charton, Lea Benabou, Marianne Tétreault-Langlois, Josselin Duchateau, Frédéric Sacher, Romain Tixier, Mélèze Hocini, Pierre Jaïs, Michel Haïssaguerre, Thomas Pambrun, Nicolas Derval

**Affiliations:** Department of Cardiac Pacing and Electrophysiology, Hopital Cardiologique du Haut-Leveque, Bordeaux University Hospital (CHU), 1 Avenue de Magellan, Pessac Cedex 33604, France; Department of Cardiac Pacing and Electrophysiology, Hopital Cardiologique du Haut-Leveque, Bordeaux University Hospital (CHU), 1 Avenue de Magellan, Pessac Cedex 33604, France; Department of Cardiac Pacing and Electrophysiology, Hopital Cardiologique du Haut-Leveque, Bordeaux University Hospital (CHU), 1 Avenue de Magellan, Pessac Cedex 33604, France; Department of Cardiac Pacing and Electrophysiology, Hopital Cardiologique du Haut-Leveque, Bordeaux University Hospital (CHU), 1 Avenue de Magellan, Pessac Cedex 33604, France; Department of Cardiac Pacing and Electrophysiology, Hopital Cardiologique du Haut-Leveque, Bordeaux University Hospital (CHU), 1 Avenue de Magellan, Pessac Cedex 33604, France; Department of Cardiac Pacing and Electrophysiology, Hopital Cardiologique du Haut-Leveque, Bordeaux University Hospital (CHU), 1 Avenue de Magellan, Pessac Cedex 33604, France; Department of Cardiac Pacing and Electrophysiology, Hopital Cardiologique du Haut-Leveque, Bordeaux University Hospital (CHU), 1 Avenue de Magellan, Pessac Cedex 33604, France; IHU Liryc, Electrophysiology and Heart Modeling Institute, University of Bordeaux, Bordeaux F-33600, France; Department of Cardiac Pacing and Electrophysiology, Hopital Cardiologique du Haut-Leveque, Bordeaux University Hospital (CHU), 1 Avenue de Magellan, Pessac Cedex 33604, France; IHU Liryc, Electrophysiology and Heart Modeling Institute, University of Bordeaux, Bordeaux F-33600, France; Department of Cardiac Pacing and Electrophysiology, Hopital Cardiologique du Haut-Leveque, Bordeaux University Hospital (CHU), 1 Avenue de Magellan, Pessac Cedex 33604, France; IHU Liryc, Electrophysiology and Heart Modeling Institute, University of Bordeaux, Bordeaux F-33600, France; Department of Cardiac Pacing and Electrophysiology, Hopital Cardiologique du Haut-Leveque, Bordeaux University Hospital (CHU), 1 Avenue de Magellan, Pessac Cedex 33604, France; IHU Liryc, Electrophysiology and Heart Modeling Institute, University of Bordeaux, Bordeaux F-33600, France; Department of Cardiac Pacing and Electrophysiology, Hopital Cardiologique du Haut-Leveque, Bordeaux University Hospital (CHU), 1 Avenue de Magellan, Pessac Cedex 33604, France; Department of Cardiac Pacing and Electrophysiology, Hopital Cardiologique du Haut-Leveque, Bordeaux University Hospital (CHU), 1 Avenue de Magellan, Pessac Cedex 33604, France; Department of Cardiac Pacing and Electrophysiology, Hopital Cardiologique du Haut-Leveque, Bordeaux University Hospital (CHU), 1 Avenue de Magellan, Pessac Cedex 33604, France; Department of Cardiac Pacing and Electrophysiology, Hopital Cardiologique du Haut-Leveque, Bordeaux University Hospital (CHU), 1 Avenue de Magellan, Pessac Cedex 33604, France; Department of Cardiac Pacing and Electrophysiology, Hopital Cardiologique du Haut-Leveque, Bordeaux University Hospital (CHU), 1 Avenue de Magellan, Pessac Cedex 33604, France; IHU Liryc, Electrophysiology and Heart Modeling Institute, University of Bordeaux, Bordeaux F-33600, France; Department of Cardiac Pacing and Electrophysiology, Hopital Cardiologique du Haut-Leveque, Bordeaux University Hospital (CHU), 1 Avenue de Magellan, Pessac Cedex 33604, France; IHU Liryc, Electrophysiology and Heart Modeling Institute, University of Bordeaux, Bordeaux F-33600, France; Department of Cardiac Pacing and Electrophysiology, Hopital Cardiologique du Haut-Leveque, Bordeaux University Hospital (CHU), 1 Avenue de Magellan, Pessac Cedex 33604, France; IHU Liryc, Electrophysiology and Heart Modeling Institute, University of Bordeaux, Bordeaux F-33600, France; Department of Cardiac Pacing and Electrophysiology, Hopital Cardiologique du Haut-Leveque, Bordeaux University Hospital (CHU), 1 Avenue de Magellan, Pessac Cedex 33604, France; IHU Liryc, Electrophysiology and Heart Modeling Institute, University of Bordeaux, Bordeaux F-33600, France; Department of Cardiac Pacing and Electrophysiology, Hopital Cardiologique du Haut-Leveque, Bordeaux University Hospital (CHU), 1 Avenue de Magellan, Pessac Cedex 33604, France; IHU Liryc, Electrophysiology and Heart Modeling Institute, University of Bordeaux, Bordeaux F-33600, France; Department of Cardiac Pacing and Electrophysiology, Hopital Cardiologique du Haut-Leveque, Bordeaux University Hospital (CHU), 1 Avenue de Magellan, Pessac Cedex 33604, France; IHU Liryc, Electrophysiology and Heart Modeling Institute, University of Bordeaux, Bordeaux F-33600, France; Department of Cardiac Pacing and Electrophysiology, Hopital Cardiologique du Haut-Leveque, Bordeaux University Hospital (CHU), 1 Avenue de Magellan, Pessac Cedex 33604, France; IHU Liryc, Electrophysiology and Heart Modeling Institute, University of Bordeaux, Bordeaux F-33600, France; Department of Cardiac Pacing and Electrophysiology, Hopital Cardiologique du Haut-Leveque, Bordeaux University Hospital (CHU), 1 Avenue de Magellan, Pessac Cedex 33604, France; IHU Liryc, Electrophysiology and Heart Modeling Institute, University of Bordeaux, Bordeaux F-33600, France

In persistent atrial fibrillation (AF), the combination of pulmonary vein isolation (PVI), vein of Marshall ethanol infusion (VOM-OH), and linear ablation at the dome, lateral mitral and cavo-tricuspid isthmuses is superior to PVI alone.^1,2^

Radiofrequency (RF) has been utilized to perform such lesion sets with an inherent risk of oesophageal injury when ablating at the posterior left atrium (LA). This risk is eliminated by pulsed field ablation (PFA), a novel and largely non-thermal form of ablation.^3^

A point-by-point contact force-sensing catheter (SmartTouch SF dual-energy, STSF-DE, Biosense Webster) capable of toggling between RF and PFA, integrated with the CARTO 3D mapping system, was developed to ablate pulmonary vein (PV)s and non-PV targets.^4^

This observational study aimed at assessing the acute efficacy of this ablation catheter. Forty consecutive patients undergoing first-time persistent AF ablation were enrolled and divided into two groups based on the ablation catheter utilized (*Figure 1A*):

1. RF-group (RF-only STSF ablation catheter)
2. Pulsed-field (PF)-group (PFA and RF with the STSF-DE ablation catheter)

The anatomical Marshall-Plan strategy,^5^ which includes PVI, lines [dome, mitral, and cavo-tricuspid isthmus (CTI)], and systematic upfront VOM-OH, was used in both groups. In the PF group, the initial attempt to block the dome was via a floor line between the lower part of the PV encirclement rather than a roof line (RF group). In the PF group, PFA was used at the posterior wall, PV carina gaps, and, in a subgroup, at the mitral isthmus; RF was used elsewhere. The last 10 patients of each group received VOM-OH after PVI and lines to elucidate the PFA effect on the mitral isthmus without the confounding effect of ethanol. Touch-ups were performed using the same energy initially employed.

**Figure 1:**
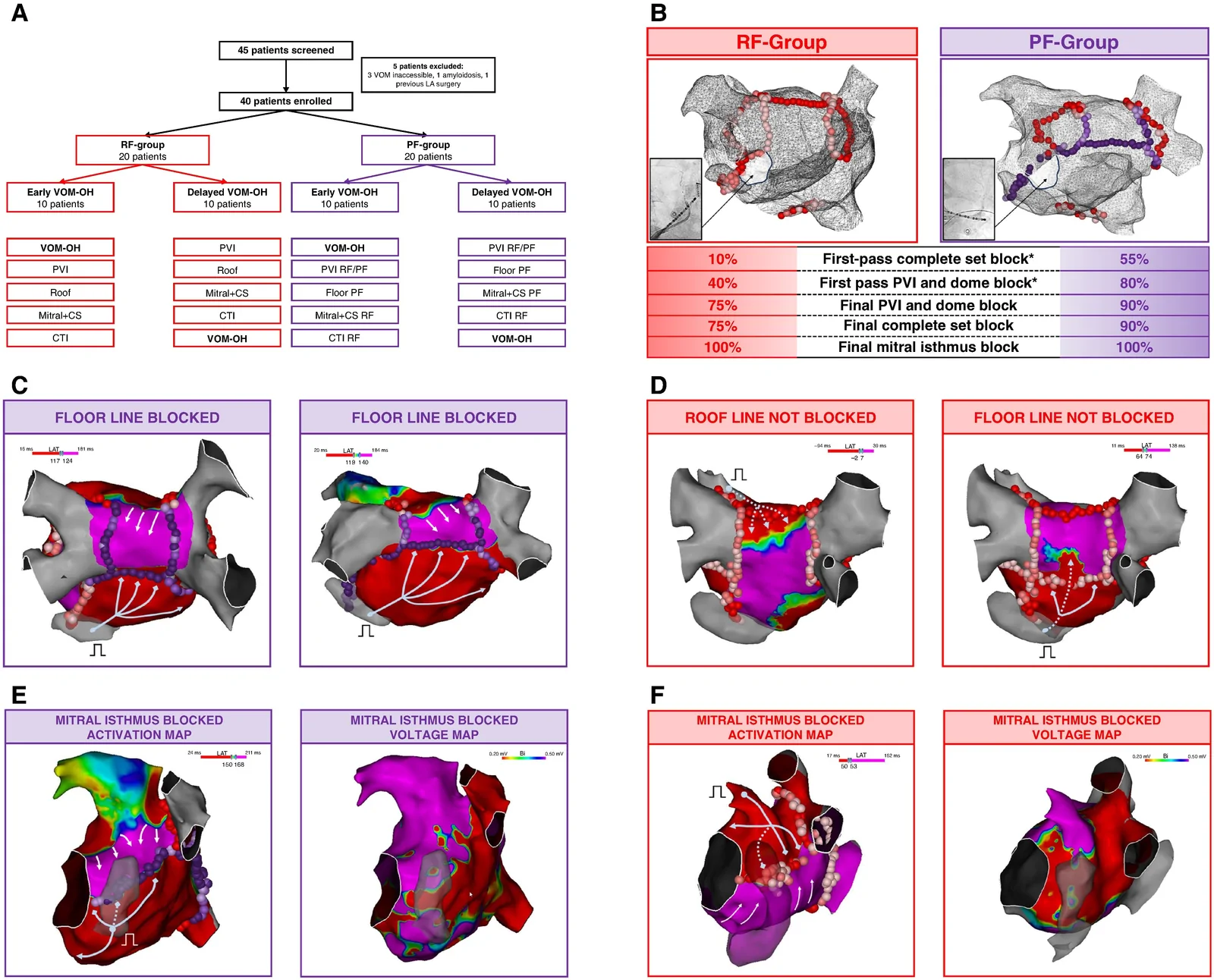
Panel *A*: Study flowchart. Panel *B*: Acute outcomes in the RF and PF group. Panel *C*: Floor line performed with PFA. The activation map during mid-CS pacing in two different patients confirmed block of the floor line; Panel *D*: Roof and floor line performed with RF. The activation map during LAA pacing demonstrated an epicardial gap across the entire roof line. A floor line was performed in order to achieve the block across the dome. The activation map during mid-CS pacing demonstrated a gap on the mid portion of the line. The dome could not be blocked despite touch-up applications. Panel *E*: PFA at the lateral mitral isthmus. The activation map during LAA pacing demonstrated block of the lateral mitral isthmus following endocardial and CS PFA and VOM-OH. On the right, the final endocardial voltage map. Panel *F*: RF at the lateral mitral isthmus. The activation map during LAA pacing demonstrated block at the lateral mitral isthmus. The block was obtained after the three steps and further touch-up ablation on the CS free wall. On the right, the final endocardial voltage map. CS, coronary sinus; LAA, left atrial appendage; PFA, pulsed field ablation; VOM-OH, vein of Marshall ethanol infusion.

Pulsed field ablation was optimized by delivering 20–24 pulses out of 24 with a minimum contact force >10 g regardless of PF index. The VisiTag size was 4 mm, and the inter-lesion distance was ≤4 mm for both RF and PFA. After a 20-min waiting period following the last ablation, a final high-density map was collected. Bidirectional block across lines was assessed using differential pacing, high-density activation map, and overlap pacing.^6^

The main objective of the study was to compare the rates of first-pass and final block of the complete set (PVI, dome, mitral isthmus, and CTI) between groups.

Continuous and categorical variables were compared using the Student’s *t*-test and the χ^2^ test, respectively. A two-sided *P*-value < 0.05 was significant.

Baseline characteristics and total procedure duration (194 ± 23 min vs. 191 ± 21 min) were similar, with 90% of procedures performed under local anaesthesia.

The rates of first-pass block of the complete set (55% vs. 10%, *P* = 0.006), first-pass PVI (85% vs. 50%, *P* = 0.041), and first-pass PVI plus dome block (80% vs. 40%, *P* = 0.022) were higher for the PF group. At the end of the procedure, the rates of complete set block (90% vs. 75%), PVI (100% vs. 100%), and PVI plus dome block (90% vs. 75%) were similar (*P* = ns).

In the delayed VOM-OH subgroup, mitral isthmus block was obtained with endocardial and coronary sinus ablation only in a single patient (10%) in each group. Following VOM-OH and touch-up ablation, mitral block was achieved in all patients.

All procedures that were carried out under local anaesthesia were successfully completed; one circumflex artery spasm occurred following PFA in the coronary sinus despite prophylactic nitrates. The main findings of the study are as follows: (i) combining PFA with RF under local anaesthesia is well tolerated and comparable to RF alone in procedure duration, final lesion set block, and acute complications. (ii) Pulsed field ablation is acutely effective at the posterior wall where epicardial components are well represented. (iii) Pulsed field ablation has similar limitations to RF at the mitral isthmus.

Although final lesion set block rates were comparable, the use of PFA had higher rates of first-pass block and required less touch-up ablation, potentially suggesting enhanced durability of the ablation set and the potential to improve clinical outcomes.^7^ Although complications were infrequent, the risk of coronary spasm with PFA should be considered when ablating close to the coronary arteries.^8^

Although end-of-procedure PVI plus dome block rates were similar, in the RF group, a floor line was needed in 40% after failing to block the dome with the roof line alone (*Figure 1D*), and the right or left carina was involved in the majority (69%) of PV gaps.

In the PF group, where the floor line was performed first, first-pass block across the dome could be achieved in 85% (*Figure 1C*), and only a single patient had a gap at the PV carina. The gaps across the mitral and dome lines were epicardial in the vast majority of patients in both the PF and RF groups, suggesting that anatomical characteristics play a pivotal role in preventing the formation of transmural lesions. The floor, where the LA wall is thinner and with less fat interposition than the roof, could be an easier target for blocking the dome. However, RF applications at this site should be down-titrated to prioritize safety, and this contributes to suboptimal lesions and gaps in lines.^9^ Pulsed field ablation can circumvent these limitations due to its inherent safety, and in the PF group, the ablation workflow was adapted by initially performing the floor line.^10^ Pulsed field ablation at this more favourable anatomical site led to 90% dome block at the end of the procedure.

Conversely, at the mitral isthmus, PFA showed the same limitations as RF with the potential to cause coronary spasms (*Figure 1E* and *F*).

A key finding of the study was that all procedures under local anaesthesia were successfully performed in the absence of muscle contractions. Only three patients needed a switch to RF for cough or pain at the inferior PVs or floor where the risk of oesophageal injury is higher.

In conclusion, anatomical ablation of persistent AF was feasible under local anaesthesia with the STSF-DE. The combination of RF and PFA was acutely effective in this cohort of patients, and the potential superiority of PFA at the posterior wall for PVI and floor line should be confirmed through further studies.

## Data Availability

Data are available upon reasonable request from the corresponding author.
